# Evaluation of a protocol for hypertonic saline administration in acute euvolemic symptomatic hyponatremia: A prospective observational trial

**DOI:** 10.4103/0972-5229.76079

**Published:** 2010

**Authors:** Emmanuel Bhaskar, Bismay Kumar, S. Ramalakshmi

**Affiliations:** **From:** Department of Medicine, Sri Ramachandra Medical College and Research Institute, Porur, Chennai, India; 1 Department of Nephrology, Sri Ramachandra Medical College and Research Institute, Porur, Chennai, India

**Keywords:** Hypertonic saline, protocol, symptomatic hyponatremia

## Abstract

**Context:**

Acute symptomatic hyponatremia is a frequent yet poorly studied clinical problem. Aims: To develop a non-weight based protocol for the treatment of acute symptomatic hyponatremia.

**Settings and Design:**

Observational study in a Multi-disciplinary Intensive Care Unit of an urban tertiary care hospital.

**Materials and Methods:**

Patients aged >18 years, admitted with euvolemic acute symptomatic severe hyponatremia (defined as serum sodium <120 meq/l with symptoms <24 hours), formed the study population. On confirmation of euvolemic status clinically and by laboratory investigations, patients were administered 100 ml of 3% NaCl over a period of 4 hours irrespective of the weight of the patient, followed by reassessment of serum Na at the end of 4 hours. The volume of hypertonic saline (in ml) required to increase serum Na by 8 meq/l was calculated using the formula: 100 × 8/increment in serum Na observed with 100 ml hypertonic saline. This volume was infused over the next 20 hours. To monitor renal water diuresis which may contribute to overcorrection, the urine specific gravity was monitored every 4 hours for sudden decrease of ≥ 0.010 from the baseline value. Measurement of serum Na was repeated if a fall in the urine specific gravity was observed and subsequently repeated every 4 hours. If no fall occurs in urine specific gravity, serum Na measurement was repeated at 12, 20 and at 24 hours (0 hour being the initiation of 100 ml hypertonic saline). The volume of infusate was adjusted if an excessive increment of serum Na (greater than 3 meq at 8 hours, 4 meq at 12 hours, 5 meq at 16 hours and 6 meq at 20 hours) was observed. Baseline characteristics were compared using chi-square test and Mann–Whitney U test.

**Results:**

58 patients formed the study cohort. The mean age was 58 years. The mean serum Na on admission was 114 meq/l. Administration of 100 ml hypertonic saline resulted in a mean increase in serum Na of 2 meq/l. The mean increase in serum Na over 24 hours was 9 meq/l and mean volume of hypertonic saline required for a serum Na increment of 8 meq/l was 600 ml.

**Conclusions:**

The non-weight based protocol with monitoring for water diuresis is reasonably an effective strategy in the treatment of acute euvolemic symptomatic hyponatremia.

## Introduction

Treatment of acute symptomatic hyponatremia is a common, yet poorly studied, clinical problem. Most teaching literature discusses the role of Androgue–Madias formula (AMF):[1] change in serum Na in meq/l with 1 L of infusate = infusate Na+ - patient’s Na^+^ /total body water + 1

However, its inconsistent clinical accuracy makes it less reliable at the bedside.[2] Water diuresis and improper assessment of hypovolemia have been proposed as possible explanations for overcorrection while applying the formula.[2] AMF is based on the principles evolved from the landmark study of Edelman *et al*, which suggested the most convincing relationship between serum sodium, potassium and total body water (TBW).[3] Edelman and colleagues arrived at their results by measuring the body weight of non-critically ill patients.[3] AMF needs assessment of TBW which is determined by lean body weight.[1] The latter is calculated based on actual body weight. Though studies have analyzed the accuracy of AMF in clinical practice, they have failed to explain how the critically ill patient’s body weight is determined on admission.[2,4] In routine clinical practice, critically ill patients do not undergo determination of weight, and drug dosages are often calculated based on clinical judgement of the weight. This limitation of measuring body weight prevents an average clinician to apply the AMF in patients with acute symptomatic hyponatremia. Furthermore, difficulty in diagnosing subclinical hypovolemia and water diuresis occurring in the course of treatment often leads to overcorrection of hyponatremia. This study was initiated to evaluate a non-weight based hypertonic saline protocol for the treatment of acute symptomatic hyponatremia in euvolemic patients, taking into consideration the methods used to identify subclinical hypovolemia and water diuresis.

## Materials and Methods

Patients aged >18 years, admitted to multi-disciplinary intensive care unit of an urban tertiary care hospital with euvolemic symptomatic severe hyponatremia of acute onset, defined as symptoms of <24 hours duration, formed the study population. The study spanned from January 2005 to July 2009. Severe hyponatremia was defined as serum Na <120 meq/l on admission. Syndrome of inappropriate antidiuresis (SIAD) was defined as a) presence of clinical euvolemia; b) serum effective osmolality <275 mOsm/kg of water; c) urine osmolality >100 mOsm/kg of water; and d) urine spot Na >40 meq/l.[5] Blood urea nitrogen (BUN) >18 mg/dl and serum uric acid >4 mg/dl indicated diagnosis of subclinical hypovolemia.[4] We excluded patients with symptoms of hyponatremia >24 hours, hypervolemia on clinical examination, clinical or subclinical hypovolemia, seizure during pre-admission or after admission, recent diuretic use, chronic kidney disease, admission creatinine >1.2 mg/dl and severe malnourished individuals. All patients were screened for hypothyroidism and adrenal insufficiency. Hypothyroidism was defined as thyroid stimulating hormone (TSH) >10 mU/l.[6] Considering the critically ill state of the study participants, adrenal insufficiency was defined as a random cortisol of <15 μg/dl or a post adrenocorticotropic hormone (ACTH) increment in cortisol <9 μg/dl after co-syntropin test.[7] Informed consent was obtained from the closest relatives of study patients. Our institutional review board approved the study.

### 

#### Protocol for Na correction

On confirmation of euvolemic status, patients were administered 100 ml of 3% sodium chloride (NaCl) over a period of 4 hours irrespective of weight of the patient. Serum Na was reassessed after completion of administration of 100 ml hypertonic saline. Based on the response to the initial infusate, the patients were divided into responders if an increment of serum Na of at least 1 meq/l was observed or non-responders if there was no increment or fall from their baseline serum sodium. The responders were then administered a volume of hypertonic saline based on the increment observed, with 100 ml hypertonic saline for the next 20 hours, with the objective of attaining a serum Na increment of 8 meq/l/24 hours from baseline. The volume of hypertonic saline (in ml) required to increase serum Na by 8 meq/l was calculated using the formula: 100 × 8 /increment in serum Na observed with 100 ml hypertonic saline

**Figure 1:**
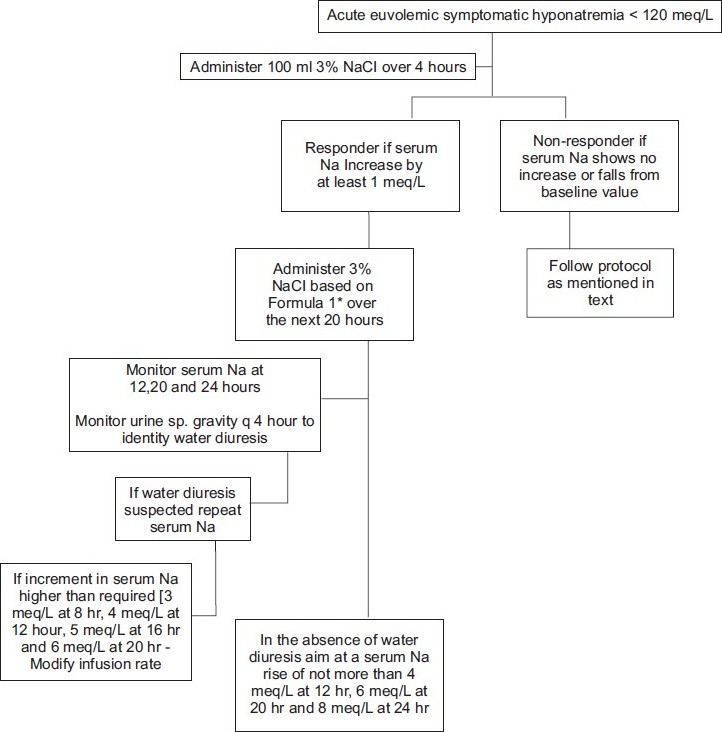
Outline of treatment algorithm *Formula 1: Infusate required in ml = (100 × 8)/increment in serum Na observed with 100 ml 3% NaCl

To monitor renal water diuresis which may contribute to overcorrection, the urine specific gravity was monitored every 4 hours for sudden decrease of ≥ 0.010 from the baseline value. In the absence of a fall in urine specific gravity, serum Na was monitored at 12, 20 and 24 hours (0 hour being the start of test fluid of 100 ml hypertonic saline). If a sudden fall in urine specific gravity was observed, an immediate assessment of serum Na was done and subsequent Na estimation was done every 4 hours. For patients who had unexpected rise (>3 meq at 8 hours, 4 meq at 12 hours, 5 meq at 16 hours and 6 meq at 20 hours) in serum Na with the calculated volume of hypertonic saline, the infusate was transiently stopped and subsequently restarted at a lower dose as calculated by our prediction formula, with close monitoring of serum Na levels every 4 hours. Free water was not administered to lower serum Na when an unexpected rise occurred. No additional intravenous fluid or oral fluid was administered during the treatment period. Though complete fluid restriction is not often recommended in the treatment of hyponatremia due to SIAD, we designed the protocol with complete fluid restriction to facilitate better laboratory assessment of renal handling of hypertonic saline and possible benefit of this approach toward adequate Na correction in the first 24 hours. Figure 1 describes an outline of treatment algorithm.

The non-responders (patients who had no increment in serum Na with 100 ml 3% saline) were subsequently administered 100 ml 3% NaCl over 1 hour followed by a serum Na reassessment. If serum Na was found to rise by at least 1 meq/l, then the protocol as explained for responders was followed, but by calculating the hypertonic saline volume using the formula: 200 × 8/increment in serum Na observed with 200 ml hypertonic saline

Patients who showed no increment in serum Na on administration of 200 ml hypertonic saline over 5 hours were administered another 100 ml of hypertonic saline over an hour and observed for an increment. If serum Na rose by at least 1 meq/l, then the protocol as explained for responders was followed but by calculating the hypertonic saline volume required to achieve a serum Na of 8 meq/l using the formula: 300 × 8/increment in serum Na observed with 300ml hypertonic saline

#### Statistical analysis

The variables were expressed in mean and range. Baseline characteristics between responders and non-responders were compared using chi-square test or Fisher exact test for categorical variables and Mann–Whitney U test for continuous variables. A *P* value of <0.05 was considered to be statistically significant.

## Results

Fifty-eight patients formed the study cohort. Mean age of the study cohort was 58 ± 12.5 years with males (*n* = 35) outnumbering females (*n* = 23). Six (10%) were chronic alcoholics, 19 (30%) were suffering from diabetes and 15 (26%) from hypertension. Five (8%) had overt hypothyroidism and 3 (5%) had relative adrenal insufficiency. Table 1 shows the presenting features of acute hyponatremia. The mean duration of symptoms was 14 hours (range 6–20 hours). Salient observations on admission and during the course of following the treatment protocol are shown in Table 2. Six (10%) had community acquired pneumonia and 15 (26%) had probable pyogenic meningitis. CT-brain showed diffuse cerebral edema in 9 (15%) patients. Forty-five (78%) patients had at least 1 meq/l of increment in serum Na following administration of 100 ml hypertonic saline; 11 (19%) patients required 200 ml hypertonic saline for initial increment in serum Na and 2 (3%) required 300 ml hypertonic saline for the initial response. Age (*P* = 0.81), sex (*P* = 0.76), alcoholism (*P* = 0.51), diabetes (*P* = 0.8) and hypertension (*P* = 0.64) showed no statistically significant difference between responders and non-responders. A fall in the urine specific gravity of ≥0.010 from baseline indicating possible water diuresis was observed in 24 (41%) patients of whom 20 had a corresponding serum Na more than expected value at that point of treatment (3 meq at 8 hours, 4 meq at 12 hours, 5 meq at 16 hours and 6 meq at 20 hours). Two (3%) patients had 1+ proteinuria at baseline and 4 (6%) had 1+ glycosuria at baseline. Fourteen (24%) patients had an increment in serum Na >8 meq/l from the baseline over 24 hours of treatment period. However, none had an increment of >12 meq/l/24 hours. Seven (12%) had a serum Na increment of <8 meq/l (minimum 6 meq/l). Forty-six (79%) patients (all 22 who had incoherent speech with mild confusion, 8 of 10 with delirium, 12 of 18 with drowsiness and 4 of 8 who were in coma) had remarkable recovery in level of consciousness defined as state of alertness with spontaneous speech and response to vocal stimuli at the end of 24 hours. The remaining two patients who presented with delirium and six patients with drowsiness improved to the extent of response to verbal stimuli but had no spontaneous speech or constant alertness, while the other four patients with coma were arousable to pain and vigorous verbal stimuli at the end of 24 hours. Plan of treatment beyond the first 24 hours was individualized based on the observation during the first 24 hours. None suffered from osmotic demyelination.

**Table 1:** Presenting features of acute hyponatremia

Symptom	No. (%)
Headache	12 (21)
Incoherent speech with mild confusion	22 (38)
Delirium	10 (17)
Drowsiness*	18 (31)
Coma**	8 (14)

* Drowsiness was defined as a state of impaired consciousness from which only vigorous external stimuli can produce arousal

** Coma was defined as a state of unconsciousness in which the patient does not respond to any type of external stimuli

**Table 2:** Salient observations on admission and during treatment

Variable	Observation mean (range)
Admission serum Na	114 (96–118) meq/l
Measured serum osmolality	230 (208–260) mOsm/kg
Measured urine osmolality	462 (310–780) mOsm/kg
Urine spot Na	70 (54–106) meq/l
BUN	12 (8–14) mg/dl
Admission urine specific gravity	1.025 (1.020–1.030)
Increment in serum Na with 100 ml 3% NaCl	2 (0–6) meq/l
Increment in serum Na over 24 hours	9 (6–12) meq/l
Volume of 3% NaCl required to increase serum Na by 8 meq/l/24 hours	600 (300–1300) ml

## Discussion

The mean increase in serum sodium of 2 meq/l with 100 ml of 3% saline observed in our study is almost double that of what may be predicted with AMF. This occurred with a volume and rate (100 ml over 4 hours) far less than the infusate required by Adrogue–Madias formula (a 50 kg male presenting with a serum sodium of 113 meq/l with acute symptoms of hyponatremia will require 120 ml of 3% saline to increase the serum Na by 1 meq/l as per Adrogue–Madias formula and the desired rate of Na correction of 0.5 meq/l/hour to 1 meq/l/hour during initial treatment mandates administration of 120 ml of 3% saline over 1–2 hours). Further, our observation that 600 ml was the mean volume of hypertonic saline required to increase the serum sodium by 8 meq/l indicates that AMF tends to overestimate the total requirement of hypertonic saline in this setting. This gets more complicated in our set-up since the patient’s body weight is often approximated and not actually measured to calculate the lean body weight and hence the TBW. Utilizing the fall in the urine specific gravity to detect water diuresis enables prediction of possible overcorrection if same dose of hypertonic saline is used despite water diuresis during the course of treatment. However, our protocol does have a lot of limitations. First, it may not be valuable in patients with marked proteinuria or glycosuria since it can influence changes in urine specific gravity, making identification of water diuresis difficult. Second, the cut-off of 0.010 fall as an indicator of water diuresis is only arbitrary and needs standardization with simultaneous measurement of urine osmolality to know the exact fall in the specific gravity which may indicate water diuresis. We were not able to compare the urine specific gravity with urine osmolality during serial measurement of the former due to cost. Since we excluded patients with seizures as presenting feature of acute hyponatremia, the protocol may not be appropriate for those patients who may need a more rapid correction. Though we observed no significant difference in the characteristics between responders and non-responders, the study was not specifically designed to identify characteristics of non-responders. A study taking into consideration factors like etiology of SIAD, hourly urine output, more frequent investigation for urinary solutes, etc., along with a larger sample of study cohort is required to identify if differences exist between responders and non-responders.

Exclusion of patients with subclinical hypovolemia using laboratory markers like BUN and serum uric acid has helped us identify euvolemic patients more accurately and hence preventing inappropriate higher dosages of hypertonic saline leading to rapid correction of serum sodium. A study evaluating accuracy of AMF used a similar approach to identify patients who were subclinically hypovolemic.[4] The initial infusate of 100 ml 3% saline over 4 hours may be a higher dose for individuals with a very low body mass, though the study cohort did not have a severely malnourished patient. Hence, we recommend against the use of this protocol in patients who are malnourished. Furthermore, the observation that 14 patients had increase in serum Na >9 meq/day despite close monitoring and guarded infusion rate highlights the complexity involved in titrating hypertonic saline in the treatment of acute symptomatic euvolemic hyponatremia.

## Conclusion

Though a common clinical problem, fear of overcorrection or rapid correction of acute symptomatic hyponatremia is an important reason for gross undercorrection of serum sodium which may have adverse consequences on clinical outcome.[8] The pathophysiology of sodium imbalance is too complex and often has significant inter-individual variations.[9] Despite being a frequent clinical issue, there is dearth of randomized control trial on management of hyponatremia. Our protocol which attempts to treat the disorder after studying the response to an initial volume of infusate and a method to check water diuresis during the course of treatment may allay the fear of overcorrection among clinicians.

Our non-weight based protocol incorporating laboratory indicators of sub-clinical hypovolemia and renal water diuresis is reasonably safe and effective in treating acute symptomatic hyponatremia. However, the protocol needs testing in a larger cohort of patients preferably in comparison with traditional approaches using a randomized controlled trial design.
